# Light-Sheet Confined Super-Resolution Using Two-Photon Photoactivation

**DOI:** 10.1371/journal.pone.0067667

**Published:** 2013-07-02

**Authors:** Francesca Cella Zanacchi, Zeno Lavagnino, Mario Faretta, Laura Furia, Alberto Diaspro

**Affiliations:** 1 Department of Nanophysics, Istituto Italiano di Tecnologia, Genova, Italy; 2 Università di degli Studi di Genova, Italy; 3 Department of Experimental Oncology, European Institute of Oncology, Milano, Italy; University of Zurich, Switzerland

## Abstract

Light-sheet microscopy is a useful tool for performing biological investigations of thick samples and it has recently been demonstrated that it can also act as a suitable architecture for super-resolution imaging of thick biological samples by means of individual molecule localization. However, imaging in depth is still limited since it suffers from a reduction in image quality caused by scattering effects. This paper sets out to investigate the advantages of non-linear photoactivation implemented in a selective plane illumination configuration when imaging scattering samples. In particular, two-photon excitation is proven to improve imaging capabilities in terms of imaging depth and is expected to reduce light-sample interactions and sample photo-damage. Here, two-photon photoactivation is coupled to individual molecule localization methods based on light-sheet illumination (IML-SPIM), allowing super-resolution imaging of nuclear pH2AX in NB4 cells.

## Introduction

The growing interest in large organisms and tissue imaging led to a rapid development in two-photon excitation microscopy [1] and light-sheet based techniques [2] [3]. The basic idea behind light-sheet based techniques relies on the selective illumination of a planar region in the focal plane coupled with a CCD based detection system. These techniques provide optical sectioning capabilities and, recently, they have been widely used to image thick biological samples. The earlier idea of orthogonally oriented illumination and detection [4] has been successfully extended to Bessel beam illumination [5] [6], structured illumination [7] [8] [9], confocal detection [10] and multiview imaging [11] [12] [13] in order to improve the resolution and imaging capability in scattering samples [14]. Within this framework, two-photon excitation [1] based approaches have been successfully implemented in light-sheet based optical configurations [15] [16] [17], thus reducing scattering effects and light-matter interactions [18]. On the other hand, in the last few years several emerging fluorescence techniques based on single-molecule localization [19] [20] [21], allowed super-resolution imaging of biological structures at molecular level [22]. The concept behind these techniques is based on the repeated detection and localization of single fluorophores with nanometer precision. The molecule sparseness, necessary for the localization, can be obtained exploiting the photoactivation process the fast conversion of photoactivatable molecules to a fluorescent state by light irradiation [23]. The super-resolution image is then obtained mapping the position of all the localized molecules, thus allowing the reconstruction of sub-cellular structures circumventing the diffraction limit. This led to a rapid development in innovative solutions providing localization-based techniques with multicolour [24] [25] and three-dimensional imaging capabilities [26] [27] [28]. Furthermore, in order to spatially confine the activation process, approaches based on temporal focusing [29] and selective plane illumination microscopy (IML-SPIM) [30] have been recently developed, allowing the 3D super-resolution imaging of whole cells and thick living cellular spheroids, respectively. All these new techniques extended the 3D imaging capabilities of localization-based methods [31] to thicker and thicker samples, thus taking a further step towards *in vivo* super-resolution imaging. Additionally, several algorithms [32], capable of improving the localization process within high-density samples [33] [34] featuring high background regimes [35], have been recently optimized. Despite this, one of the main limitations to super-resolution imaging of large samples is still represented by light-sample interactions and refractive index inhomogeneities since absorption and scattering effects may induce photon losses and spreading respectively. In the last decades, two photon excitation has been proven to be a golden standard to improve imaging depth capabilities and to reduce the scattering effects thanks to the use of higher wavelengths [36]. Furthermore, the two photon process has been demonstrated to be a suitable approach to perform photoactivation experiments using photoactivatable dyes or fluorescent proteins such as photoactivatable green fluorescent protein (PaGFP) [37]. In this case, after photoactivation, the excitation spectrum shifts maintaining the very same emission spectrum of the single-photon case for the native and photoactivated protein [38].

Within this framework, two-photon (2P) photoactivation may thus be a key tool for improving the performance of localization-based super-resolution techniques, allowing higher imaging depths to be achieved and improving the confinement of the photoactivation process in thick samples. 2P process followed by widefield excitation was initially implemented in order to perform 2D super-resolution [39] [40] and 3D photoactivation localization microscopy (PALM) of “whole” cells [29], confining the photoactivation process and thus alleviating background noise. Within this framework, two-photon photoactivation can provide benefits to the localization performance of recently developed super-resolution imaging methods for thick samples such as IML-SPIM. In particular, when localization based super-resolution imaging is performed within selective plane illumination architecture, tissue-induced distortions and scattering effects may cause an enlargement of the light-sheet intensity distribution, thus generating an undesired photoactivation of molecules outside the light-sheet volume. Additionally, when super-resolution imaging of live biological samples is performed, two-photon photoactivation makes it possible to reduce the photo-damage compared with UV-induced activation. This paper demonstrates super-resolution obtained by two-photon photoactivation confined in a light-sheet optical illumination scheme.

## Results

**Figure 1:**
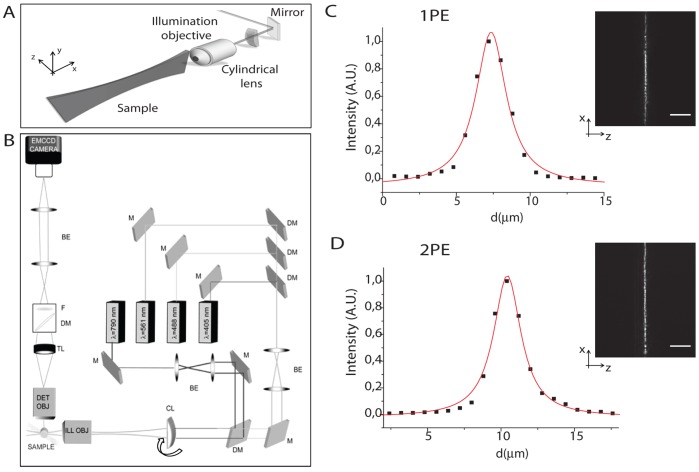
Schematic representation of the SPIM illumination geometry (1A). IML-SPIM optical system adapted for two-photon photoactivation (1B). M mirrors, DM dichroic mirrors, BE beam expander, CL cylindrical lens, TL tube lens, F bandpass filter. Either a sample or a mirror for alignment can be alternatively inserted into the holder mounted on the motorised stages. Direct observation of the light-sheet illumination intensity distribution created by the cylindrical lens is achieved by inserting a plane mirror into the sample holder, oriented at 45 degrees with respect to the illumination axis, acquiring the reflected light on the EMCCD camera. Illumination light-sheet distributions with a comparable thickness were chosen for single-photon and two-photon excitation (1C insets). The illumination intensity (fig. 1C insets) can be described by an elliptical gaussian-lorentzian distribution and its thickness can be obtained by performing a fitting procedure: examples of sample curves and the relative fits are shown in fig. 1C for single-photon excitation (R^2^≈0.99) and two-photon excitation (R^2^≈0.98)). Scale Bar = 50 µm. Objective: Leica HCX APO L U–V–I 20×.

When imaging of scattering sample is performed using conventional SPIM, where illumination in the visible range is used, potential spreading of the exciting photons [18] and the consequent enlargement of the effective excitation volume can occur [41]. Within this scenario, in order to investigate the advantages of 2P photoactivation, we first assessed the effects induced by light-sample interactions on the effective excitation volume obtained using similar illumination conditions both for single- and two-photon regime. The light-sheet is created using a cylindrical lens (see fig. 1A) according to the conventional selective plane illumination configuration [2] shown in fig. 1B. The basic idea behind selective plane illumination microscopy (SPIM) relies on the confined illumination within a thin plane orthogonally oriented to the detection axis. The illumination and the detection paths are thus decoupled and only a planar region in the focal plane is excited. The light-sheet is generated using a cylindrical lens and a low magnification objective in order to obtain the suitable light sheet dimension and to reduce aberrations. The sample is held in a water chamber and is moved within the focus of the detection lens in order to perform optical sectioning.

First, we characterize the illumination intensity distribution and the light-sheet dimension imaging the illumination beam both in the visible and in the IR range. A direct measurement of the illumination light-sheet thickness can be made by reflection imaging of the incident light: a plane mirror is inserted in the sample holder and oriented at 45 degrees with respect to the illumination axis and the reflected light is acquired on the CCD camera (as shown in fig. 1B). Calibrated illumination conditions were set for single-photon (488nm) and for two-photon (790 nm) regime in order to obtain illumination light sheet distributions endowed with a comparable thickness. A fitting procedure (fig. 1C) and the FWHM calculation provide the thickness of the illumination light-sheet, which proves to be (2.6±0.4)µm in the linear configuration (fig. 1C) and (2.8±0.4)µm in the non-linear configuration (fig. 1D). Mean values and the statistical errors are calculated over 20 different repeated measurements.

**Figure 2:**
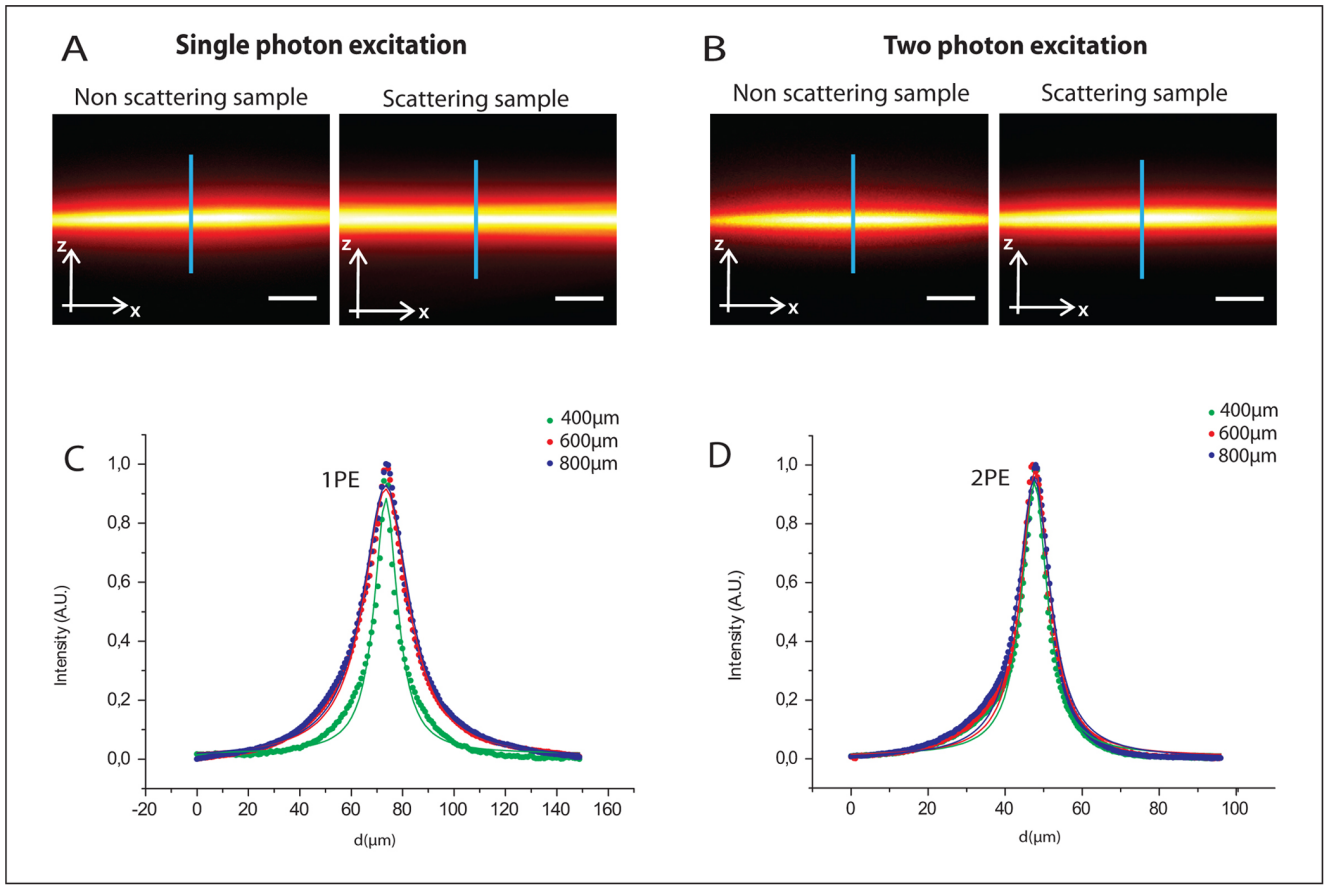
Single-photon (2A) and two-photon excitation (2B) cross-sectional views of light-sheet intensity distribution in scattering phantom samples (µ_s_ = 50mm ^−1^) and quite transparent (µ_s_ = 5mm^−1^) phantom samples. The excitation intensity distribution can be characterized measuring the thickness of the axial profile along the detection axis z (blue line). Imaging of the effective light-sheet fluorescence intensity distribution in scattering phantom samples (µ_s_ = 50 mm^−1^) and quite transparent (µ_s_ = 5 mm^−1^) phantom samples is performed at different illumination depths: 400 µm, 600 µm, and 800 µm. Intensity profiles of the XZ cross-section (blue line in fig. 2A, 2B) within the scattering phantom sample under 1PE (2C) and 2PE regime (2D). A greater enlargement of the thickness of the effective excitation distribution is evident for 1PE compared to 2PE. Scale Bar = 20 µm. Objective: Leica HCX APO L U–V–I 40×. Excitation wavelengths used: 488 nm and 790 nm respectively.

**Figure 3:**
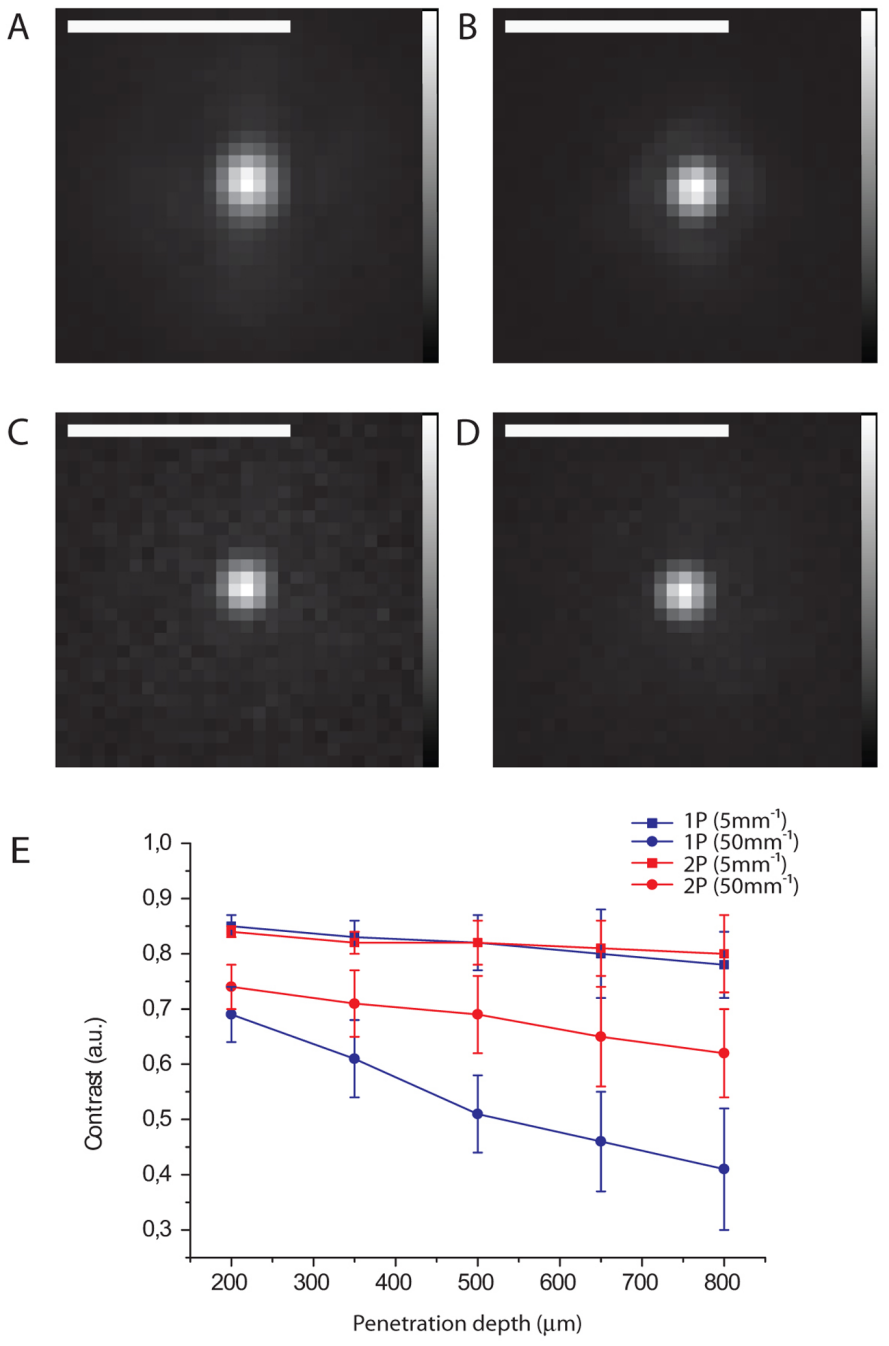
Contrast measurements are performed by imaging fluorescent beads (1 µm) in a transparent sample in both single-photon excitation (fig. 3A) and two-photon excitation (fig. 3B). Similar measurements were carried out in an immobile scattering phantom sample (µs = 50mm^−1^) for both 1PE (fig. 3C) and 2PE (fig. 3D). Contrast vs. illumination depth (3E) in scattering (square) and quite transparent (circles) phantom samples in both the linear (black line) and non-linear (red line) configurations. Error bars show the statistical error over 20 measurements. Excitation wavelengths used: 488 nm and 740 nm respectively. Detection objective: Leica HCX APO L U–V–I 40×. Scale Bar = 3 µm.

Thereafter, the excitation intensity distribution can be characterized by fluorescence SPIM imaging both in non scattering and highly scattering samples, in order to investigate effects due to incident scattered photons. The effective volume where 1P and 2P excitation occurs within the light-sheet geometry can be studied by imaging XY and XZ cross-sectional views of the fluorescence intensity distribution on an immobile fluorescent sample (materials and methods). A rotation of the cylindrical lens allows to switch easily between the XY and the XZ views. The XZ cross sectional view of the fluorescence intensity distribution is imaged on a CCD camera with the cylindrical lens rotated by 90^0 ^with respect to conventional imaging orientation both for single-photon excitation (see fig. 2A) and for two-photon excitation (see fig. 2B). The illumination source used for 2PE is a tunable Ti:Sapphire laser, Chameleon ULTRA II, Coherent Inc. (pulse durations of ∼140 fs and repetition rate of 80 MHz across the tuning range (680–1060 nm). The non-linear dependence of the emitted fluorescent signal on excitation power was verified experimentally (data not shown) in order to demonstrate the 2PE regime despite the low photon density configuration provided by the light-sheet. In order to evaluate sample-induced distortions affecting the excitation volume, mainly due to scattering effects, we analysed the XZ profile of the fluorescence intensity distribution in uniform samples with different scattering coefficients. To this end, scattering phantom samples, capable of mimicking the optical properties of transparent biological samples (scattering coefficient µ_s_ = 5 mm^−1^) and mammalian cell tissue (µ_s_ = 50 mm^−1^) have been used [42]. Changes in fluorescence intensity distribution due to the increased illumination path length in different scattering phantom samples makes it possible to evaluate the distortions induced by light-sample interaction on the light-sheet shape. In fact, the more the light travels through the sample, the more evident the effects on the effective light-sheet excitation volume are. Imaging of the XZ cross-section of the excitation intensity distribution corresponding to different illumination depths (400, 600 and 800 µm within the sample) is performed (at a constant detection depth of 150 µm). The FWHM of the intensity profile along the axial path (see blue line in figs. 2A, 2B) allows us to evaluate the distortion of the effective excitation distribution damaging the optical sectioning capability. In fact, the experimental profile of the effective light-sheet distribution in a uniform scattering sample (µ_s_ = 50mm^−1^) shows a more pronounced distortion in the linear configuration (see fig. 2C) than in the non-linear configuration (see fig. 2D) as a function of the illumination depth increase. The FWHM values of the excitation intensity profiles, with the illumination light-sheet thickness being equal for single- and two-photon excitation, are obtained using a gaussian-lorentzian fitting procedure, both for single-photon excitation (488nm) and two-photon excitation (790nm), at different illumination depths (table 1). Average FWHM values are statistically obtained over 20 samples and the mean values of the adjusted R-square are shown in table 1 for highly scattering samples. When the light-sheet position is shifted from 400 µm to 800 µm deep within the scattering sample (µ_s_ = 50 mm^−1^) the thickness of the effective excitation distribution increases: a 73.5% increment in the linear configuration and a 23.7% increment in the non-linear configuration are reported. Considering the lower scattering phantom sample (µ_s_ = 5 mm^−1^), the FWHM of the light-sheet intensity profile shows a closer thickness increase (17% for 1PE and 18% for 2PE), while varying the illumination depth (from 400 µm to 800 µm). The corresponding values (corresponding to the mean values over 20 repeated measurements and the statistical errors) are reported in table 1 both for the low scattering and the high scattering regimes. The observed distortion of the effective fluorescence light sheet profile and the increased thickness of the excitation intensity distribution can be considered as a direct consequence of the photon spreading induced by the scattering properties of the sample. This effect is minimised when moving to two-photon excitation where the Gaussian shape is well preserved and the excitation process is confined better especially in highly scattering samples. The reduced axial confinement of the light-sheet excitation affects the optical sectioning capabilities of the SPIM system and induces a higher background when the illumination depth increases (fig. 3). This effect is less evident when 2P light sheet regime is used resulting in an image contrast improvement. Contrast measurements are performed by imaging fluorescent beads embedded both in transparent (µ_s_ = 5mm^−1^) (fig. 3A, 3B) and in scattering (µ_s_ = 50mm^−1^) (fig. 3C, 3D) phantom samples at different illumination depths (from 200 µm to 800 µm). The average value of the contrast 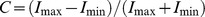 is calculated both for 1PE (λ_exc_ = 488nm) (fig. 3A, 3C) and 2PE (λ_exc_ = 740nm) (fig. 3B, 3D), where I_max_ is the peak intensity corresponding to fluorescent beads and I_min_ is the mean value of the background calculated in a far region from any fluorescent marker. The dependence of the contrast on the illumination depth (fig. 3E) in scattering phantom samples (square) shows higher contrast values for 2PE (red line) than 1PE (black line). On the other hand, contrast is comparable in the linear and non-linear regime when measurements are performed under low scattering conditions, i.e. in a quite transparent sample (circles). So, as a starting point we demonstrated a more precise confinement of the light sheet volume within scattering samples under 2P regime and the consequent contrast improvement.

**Table 1:** Excitation distribution intensity profiles.

Penetrationdepth (µm)	1PE Low scattering (5 mm^−1^)	1PE High scattering (50 mm^−1^)	2PE Low scattering (5 mm^−1^)	2PE High scattering (50 mm^−1^)
400 µm	8.2±0.6 µm	12.6±0.8 µm R^2^ = 0.97	7.2±0.4 µm	8.2±0.5 µm R^2^ = 0.98
600 µm	8.6±0.5 µm	20.7±1.3 µm R^2^ = 0.99	7.6±0.6 µm	9.2±0.8 µm R^2^ = 0.99
800 µm	9.6±0.6 µm	21.9±1.0 µm R^2^ = 0.99	8.5±0.9 µm	10.2±1.0 µm R^2^ = 0.98

Summary of mean values of the FWHM of light-sheet intensity profiles along the axial path in single- and two-photon excitation while increasing the illumination path. A greater thickness of the effective excitation distribution is observed for single-photon excitation (488 nm) compared with two-photon excitation (790 nm) when the light is focused in a highly scattering (50 mm^−1^) samples (the mean adjusted R^2^ is indicated). For measurements performed in the low scattering regime the adjusted R^2^ values are higher than 0.99. The imaging depth is set at 150 µm.

**Figure 4:**
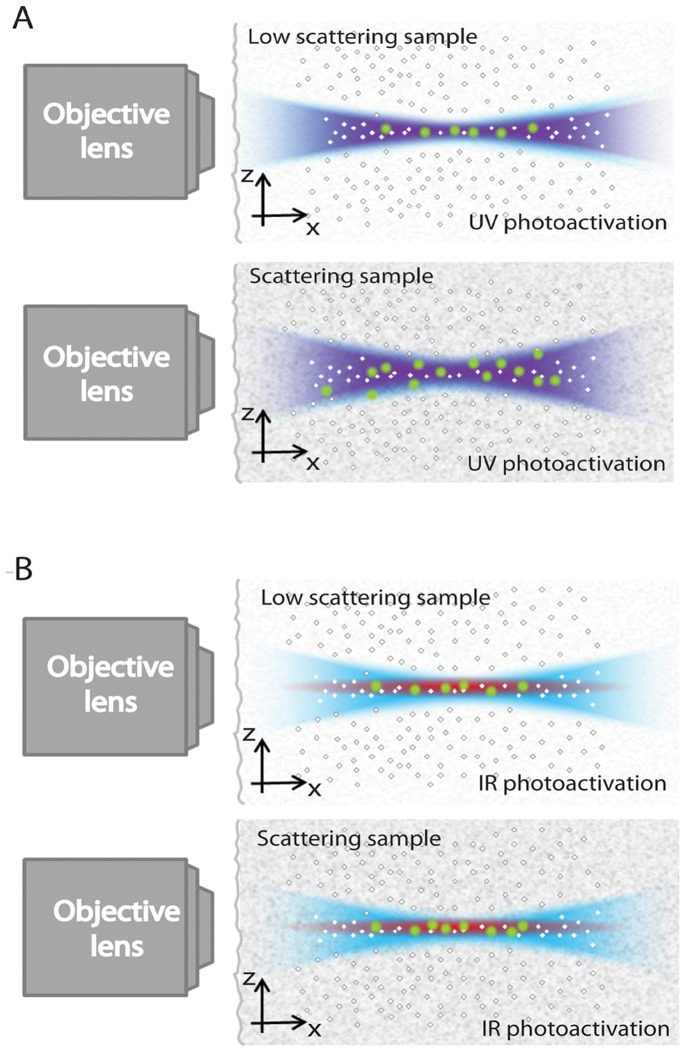
Schematic diagram of the photoactivation and excitation volumes in IML-SPIM: photoactivation performed using UV light (violet) exhibits a lower spatial confinement (4A) in the presence of scattering samples compared to two-photon (red) induced photoactivation (4B). Readout, performed in the VIS range (light blue), is used to a sparse subset of activated molecules (green) in a confined region.

This preliminary analysis on the effects provided by a 2PE approach within a selective plane illumination scheme suggests that single molecule localization can benefit from the confined excitation process demonstrated above. IML-SPIM can be promoted by confining the photoactivation process towards applications to imaging of large scattering samples in depth as a perspective. In this framework, fig. 4 represents the XZ cross-sections of the light-sheet photoactivation volumes both for single-photon (see fig. 4A) and two-photon induced activation (see fig. 4B). When photoactivation in scattering samples is performed using UV-VIS wavelengths, the thickness of the effective photoactivation volume increases and the confinement of the molecules involved in the image formation process is reduced, as shown in the schematic diagram in fig. 4A. On the other hand, when priming the photoactivation process in a confined volume using 2PE, molecules arising from a more homogeneous light-sheet distribution can be localized when imaging large scattering samples (see fig. 4B). The confinement of the photoactivation process and the improved contrast in scattering samples, direct consequences of the preserved light-sheet spatial distribution, suggests that two-photon process can be a useful solution for photoactivation in IML-SPIM.

**Figure 5:**
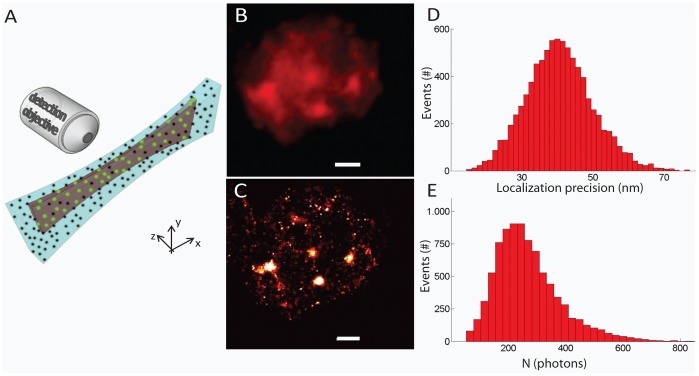
Schematic diagram (5A) of the photoactivation (dark violet illumination volume) and readout light-sheet intensity distribution (light blue illumination volume). Only molecules within the activation volume are photoactivated when using 2P photoactivation (green dots). Super-resolution imaging by IML-SPIM based on 2P photoactivation of a nuclear protein (pH2AX) in NB4 cells (5C) with a mean localization precision of 40 nm. Comparison between conventional SPIM (5B) and 2PE IML-SPIM (5C) images of nuclear pH2AX stained with a photoactivatable dye FLIP565 (Abberior). The super-resolution image is obtained acquiring 5000 frames in order to have a sufficient amount of localized molecule to reconstruct the final image. The conventional SPIM image is obtained by adding the total signal from all the frames (5B). Exposure time for each frame: 30ms. Total acquisition time: 2.5 min. Cells were embedded 200 µm within the agarose gel. The activation and excitation laser wavelengths were 760 nm (intensities about 35–200 kW/cm^2^) and 561 nm (at intensity 9 kW/cm^2^), respectively. The number of photons/molecule collected and the localization precision obtained in E and D respectively. Scale bar = 1 µm. Objective lens: CFI Plan 100× W, NA 1.1.

Here, we will focus on the demonstration of IML-SPIM imaging of subcellular structures exploiting 2P photoactivation. 2P photoactivation reduces scattering effects and preserves the activation volume shape, thus improving the confinement of the photoactivation process in scattering samples. In such a configuration, only the photoactivated molecules within the light-sheet volume are turned on (as shown in the schematic diagram in fig. 5A) and imaged by IML-SPIM. Following the experimental evidence and the previous considerations, we demonstrate, for the first time, individual molecule localization in a standard light-sheet regime taking advantage of 2P photoactivation at 760 nm. Individual molecule localization after 2P photoactivation is exploited to perform super-resolution imaging of the phospho-histonH2A.X (pH2AX) in NB4 cells. Immunostaining of NB4 cells with photoactivatable dyes (FLIP565, Abberior), previously used in literature for single-molecule detection and 2P photoactivation [43], was performed. The comparison between the conventional SPIM imaging (fig. 5B) of the spatial distribution of the nuclear pH2AX and the corresponding super-resolution imaging with 2P photoactivation (fig. 5C) shows the imaging improvement provided by 2P IML-SPIM. In the super-resolution experiment the readout was obtained exciting the photoactivated molecules at 561 nm. We used a CW readout laser (561 nm) for imaging and an IR pulsed laser at 760 nm to prime photoactivation, both of which ran continuously during the entire experiment (5000 frames). Prior to 2P photoactivation, an initial high-power illumination phase at 561nm is performed in order to bleach all the previously photoactivated molecules in the sample. The intensities used for 2Pn photoactivation and single photon readout were 50–200kW/cm^2^ and 9KW/cm^2^ respectively. The photactivation power is gradually increased during the entire experiment in order to control the number of active molecules/frame. The relatively low power used for photoactivation and the short pulse duration used allow to reduce thermal effects and sample instabilities. To analyse the IML-SPIM images, we used an established localization approach based on the procedure previously indicated for super-resolution imaging through fluorescence photoactivation localization microscopy [21]. We collected photons from individual fluorescent molecules achieving a mean lateral localization precision of 40 nm. The localization precision achieved depends on the number of photons/molecule collected and is calculated according the previous work by Mortensen and colleagues [44]. Histograms show the distribution of the localization precision reached (fig. 5D) and the number of photons/molecule collected (fig. 5E), thus demonstrating that 2P photoactivation IML-SPIM provides an efficient single molecule mapping.

## Discussion

In this work, we demonstrated for the first time the utilization of 2P photoactivation in a light-sheet architecture towards IML-SPIM super-resolution imaging. The relevance of such an approach lies on coupling the benefits of 2PE imaging when dealing with highly scattering objects and super-resolution capabilities already demonstrated for 1PE IML-SPIM. First, the evaluation of the effects induced by light-sample interactions and a comparison between the performances of 1P and 2P regimes were performed. Particular attention has been addressed to the distortions induced on the effective excitation distribution when imaging non-transparent samples in depth. Experiments showed a distortion of the effective fluorescence light-sheet profile and an enlarged excitation intensity distribution due to the photon spreading induced by the scattering properties of the sample in the linear regime. The light sheet confinement and the improved contrast shown under 2P regime in thick scattering samples suggests this technique as a promising solution to improve the imaging capabilities of light-sheet based super-resolution techniques. Following the demonstration of the improved performances of the 2P based approach in terms of the homogeneity of the light sheet excitation, we showed IML-SPIM imaging based on 2P photoactivation of nuclear pH2AX in NB4 cells reaching a 40 nm localization precision. The 2P photoactivation IML-SPIM has great potential for the super-resolution imaging of complex biological specimens thanks to the reduction of scattering effects when realizing the sparse molecules photoactivation in depth. 2P-IML-SPIM can be used to browse through living or fixed samples with limited photodamage, thus targeting specific events for super-resolution analysis with the unique penetration depth provided by infrared non-linear interactions.

## Methods

### Immobile Fluorescent Sample

The derived fluorescence emission intensity distribution was collected by imaging FITC Dextrans molecules (1 mg/10 ml, m.w. 500KDa) embedded in 1.5% agarose gel.

### Phantom Scattering Sample

Fluorescent phantom samples with calibrated scattering coefficients were made by entrapping FITC dextrans (1 mg/10 ml, m.w. 500 KDa, Sigma Aldrich) in 1.5% low-melting-point agarose (Sigma Aldrich, low melting) diluted in 1× PBS (pH 7.4) and embedding different amounts of non-fluorescent polystyrene microspheres (d = 1.03 µm, Polysciences) in the agar solution in order to control the scattering properties of the sample. Scattering phantom samples capable of mimicking the mean optical properties of transparent biological samples (5 mm^−1^) and mammalian cell tissue (50 mm^−1^) were obtained using concentrations of 3.64, and 36.4 particles/µl (Mourant, et al., 1998) respectively. The sample was embedded in agarose gel and enclosed in a glass capillary (Socorex capillary tube, volume 60–100 ml, diameter 1mm).

Contrast measurements in a phantom scattering sample (1 µm sized scattering beads were embedded in 1.5% agarose gel resulting in a controlled scattering coefficient of 50mm^−1^) were performed by imaging sub-resolved fluorescent beads (2 mg/ml (W/V) Invitrogen FluoSphere, 173 nm diameter).

### Two-photon IML-SPIM Optical Set Up

The optical system used (fig. 1b) is based on the IML-SPIM [29] architecture adapted for two-photon photoactivation. The illumination unit is based on a conventional SPIM configuration [2] in which a cylindrical lens (Thorlabs LJ1653R-B, *f = *200 mm) focuses the light in the back focal plane of an objective lens (Nikon Plan, 10×, NA 0.3) to produce a thin light-sheet (Fig. 1a).

Illumination can be generated in the visible range by solid state diode lasers operating at different wavelengths (Coherent Cube 405 nm–100 mW, Coherent Sapphire OPSL 488 nm, 200 mW, Coherent Sapphire OPSL 561 nm, 200 mW). The laser beam from an ultrafast Ti:sapphire IR laser (Chameleon UltraII; Coherent) was collinearly combined to perform 2PE. Different laser beam expanders (providing 2× and 1.66× magnifications respectively) were used to create comparable illumination light-sheets for 1PE (Thorlabs AC254–030–A–ML, focal length *f*1 = 30 mm and Thorlabs AC254–060ML, *f*2 = 60 mm) and for 2PE (Thorlabs AC254-030-B, focal length *f*1 = 30 mm and Thorlabs AC254-050-B, *f*2 = 50mm). A bank of dichroic mirrors (Chroma Z405RDC, Chroma Z488RDC, Chroma Z561RDC and Semrock FF670 Sdi01) was used to combine visible and IR laser beams into a collinear path to produce overlapping light-sheets of multiple wavelengths.

Imaging is performed using a back-illuminated electron-multiplying charge-coupled device (CCD) camera (Andor Ixon DU–897E–CS0BV), a regular tube lens (Thorlabs AC254200–A–ML), and water-dipping objective lenses (HCX APO L U–V–I 20× NA 0.5, HCX APO L U–V–I 40× NA 0.8 or CFI Plan 100× W NA 1.1, with spherical aberration correction). Dichroic mirrors (Chroma, T505LP and T570LP), band-pass dichroic filters (Semrock Brightline Fluofilter 525/50 BP and Semrock Brightline FluoFilter 607/70 BP) and short pass filter (Chroma, Filter SP680) made it possible to reject excitation light and to select the fluorescence signal.

### Cell Culture and Immunofluorescence of NB4 Cells

NB4 cell line was established from a human Acute Promyelocytic Leukemia (APL) [45]. NB4 cells were grown in RPMI 1640 medium containing 10% FBS, 2 mM glutamine, 50 ng/ml Penicillin/Streptomycin (Lonza) at 37°C in 5% CO_2_ and fixed in 2% formaldehyde for 10 minutes at 4°C. Fixed NB4 cells were washed in PBS and permeabilised for 10 minutes in a permeabilisation buffer containing 0.1% Triton X-100 (vol/vol) in PBS. The cells were then incubated for 30 minutes in a blocking solution, 5% BSA (wt/vol) in PBS, and then incubated for 1 hour at room temperature with primary antibodies anti phospho-histonH2A.X (pH2AX )Biolegend) diluited in the blocking solution. The cells were rinsed 3 times in PBS and incubated for 1 hour at room temperature with secondary antibody anti mouse FLIP565 (Abberior). The cells were rinsed 3 times in PBS once again.

## References

[1] Denk W, Strickler J, Webb WW (1990) Two photon laser scanning fluorescence microscopy. Science 248 (4951): 73–76. 10.1126/science.2321027 2321027

[2] Huisken J, Swoger J, Del Bene F, Wittbrodt J, Stelzer EHK (2004) Optical sectioning deep inside live embryos by selective plane illumination microscopy. Science 305 (5686): 1007–1009. 10.1126/science.1100035 15310904

[3] Keller PJ, Schmidt AD, Wittbrodt J, Stelzer EHK (2008) Reconstruction of zebrafish early embryonic development by scanned light sheet microscopy. Science 322(5904): 1065–1069. 18845710 10.1126/science.1162493

[4] Siedentopf H, Zsigmondy R (1903) Über Sichtbarmachung und Größenbestimmung ultramikroskopischer Teilchen mit besonderer Anwendung auf Rubingläser. Ann. Phys. 10: 1–39.

[5] Planchon TA, Gao L, Milkie DE, Davidson MW, Galbraith JA, Galbraith CG, Betzig E (2011) Rapid three-dimensional isotropic imaging of living cells using Bessel beam plane illumination. Nat Methods 8 (5): 417–423. 10.1038/nmeth.1586 PMC3626440 21378978

[6] Fahrbach FO, Rohrbach A (2012) Propagation stability of self-reconstructing Bessel beams enables contrast-enhanced imaging in thick media. Nat Commun 3: 632. 22252556 10.1038/ncomms1646

[7] Keller PJ, Schmidt AD, Santella A, Khairy K, Bao Z, et al (2010) Fast, high-contrast imaging of animal development with scanned light sheet-based structured-illumination microscopy. Nat Methods 7(8): 637–642. 20601950 10.1038/nmeth.1476 PMC4418465

[8] Mertz J, Kim J (2010) Scanning light-sheet microscopy in the whole mouse brain with HiLo background rejection. J Biomed Opt 15 (1): 16–27, 2010. 10.1117/1.3324890 PMC2917465 20210471

[9] Gao L, Shao L, Higgins CD, Poulton JS, Peifer M, et al (2012) Noninvasive imaging beyond the diffraction limit of 3D dynamics in thickly fluorescent specimens. Cell 151(6): 1370–1385. 23217717 10.1016/j.cell.2012.10.008 PMC3615549

[10] Silvestri L, Bria A, Sacconi L, Iannello G, Pavone FS (2012) Confocal light sheet microscopy: micron-scale neuroanatomy of the entire mouse brain. Opt Express, 20 (18): 20582–20598. 10.1364/OE.20.020582 23037106

[11] Verveer PJ, Swoger J, Pampaloni F, Greger K, Marcello M, et al (2007) High-resolution three-dimensional imaging of large specimens with light sheet-based microscopy. Nat Methods 4(4): 311–313. 17339847 10.1038/nmeth1017

[12] Tomer R, Khairy K, Amat F, Keller PJ (2012) Quantitative high-speed imaging of entire developing embryos with simultaneous multiview light-sheet microscopy. Nat Methods 9(7): 755–763. 22660741 10.1038/nmeth.2062

[13] Krzic U, Gunther S, Saunders TE, Streichan SJ, Hufnagel L (2012) Multiview light-sheet microscope for rapid in toto imaging. Nat Methods 9(7): 730–733. 22660739 10.1038/nmeth.2064

[14] Ntziachristos V (2010) Going deeper than microscopy: the optical imaging frontier in biology Nat Methods. 7(8): 603–614. 10.1038/nmeth.1483 20676081

[15] Truong TV, Supatto W, Koos DS, Choi JM, Fraser SE (2011) Deep and fast live imaging with two-photon scanned light-sheet microscopy Nat Methods. 8(9): 757–760. 10.1038/nmeth.1652 21765409

[16] Palero J, Santos SICO, Artigas D, Loza-Alvarez P (2010) A simple scanless two-photon fluorescence microscope using selective plane illumination Opt Express. 18(8): 8491–8498. 10.1364/OE.18.008491 20588695

[17] Zanacchi FC, Lavagnino Z, Pesce M, Difato F, Ronzitti E, et al.. (2011) Two-photon fluorescence excitation within a light sheet based microscopy architecture Proc. of SPIE 7903.

[18] Lavagnino Z, Zanacchi FC, Ronzitti E, Diaspro A (2013) Two-photon excitation selective plane illumination microscopy (2PE-SPIM) of highly scattering samples: characterization and application Opt Express. 21(5): 5998–6008. 10.1364/OE.21.005998 23482168

[19] Betzig E, Patterson GH, Sougrat R, Lindwasser OW, Olenych S, et al (2006) Imaging intracellular fluorescent proteins at nanometer resolution Science. 313(5793): 1642–1645. 10.1126/science.1127344 16902090

[20] Rust MJ, Bates M (2006) Zhuang× (2006) Sub-diffraction-limit imaging by stochastic optical reconstruction microscopy (STORM). Nat Methods 3(10): 793–795. 16896339 10.1038/nmeth929 PMC2700296

[21] Hess ST, Girirajan TPK, Mason MD (2006) Ultra-high resolution imaging by fluorescence photoactivation localization microscopy. Biophys J 91(11): 4258–4272. 16980368 10.1529/biophysj.106.091116 PMC1635685

[22] Huang B, Bates M (2009) Zhuang× (2009) Super-resolution fluorescence microscopy. Annu Rev Biochem 78: 993–1016. 19489737 10.1146/annurev.biochem.77.061906.092014 PMC2835776

[23] Patterson GH, Lippincott-Schwartz J (2002) A photoactivatable GFP for selective photolabeling of proteins and cells. Science 297(5588): 1873–1877. 12228718 10.1126/science.1074952

[24] Bates M, Huang B, Dempsey GT (2007) Zhuang× (2007) Multicolor super-resolution imaging with photo-switchable fluorescent probes. Science 317(5845): 1749–1753. 17702910 10.1126/science.1146598 PMC2633025

[25] Gunewardene MS, Subach FV, Gould TJ, Penoncello GP, Gudheti MV, et al (2011) Superresolution imaging of multiple fluorescent proteins with highly overlapping emission spectra in living cells. Biophys J 101(6): 1522–1528. 21943434 10.1016/j.bpj.2011.07.049 PMC3177078

[26] Huang B, Jones SA, Brandenburg B (2008) Zhuang× (2008) Whole-cell 3D STORM reveals interactions between cellular structures with nanometer-scale resolution Nat Methods 5. (12): 1047–1052. 10.1038/nmeth.1274 PMC2596623 19029906

[27] Prasanna SR, Thompson MA, Biteen JS, Lord SJ, Liu N, et al (2009) Three-dimensional, single-molecule fluorescence imaging beyond the diffraction limit by using a double-helix point spread function Proc Natl Acad Sci. 106(9): 2995–2999. 10.1073/pnas.0900245106 PMC2651341 19211795

[28] Juette MF, Gould TJ, Lessard MD, Mlodzianoski MJ, Nagpure BS, et al (2008) Three-dimensional sub-100 nm resolution fluorescence microscopy of thick samples. Nat Methods 5(6): 527–529. 18469823 10.1038/nmeth.1211

[29] York AG, Ghitani A, Vaziri A, Davidson MW, Shroff H (2011) Confined activation and subdiffractive localization enables whole-cell PALM with genetically expressed probes. Nat Methods 8(4): 327–333. 21317909 10.1038/nmeth.1571 PMC3073501

[30] Zanacchi FC, Lavagnino Z, Donnorso MP, Del Bue A, Furia L, et al (2011) Live-cell 3D super-resolution imaging in thick biological samples. Nat Methods 8(12): 1047–1049. 21983925 10.1038/nmeth.1744

[31] Huang B (2011) An in-depth view Nat Methods. 8(4): 304–305. 10.1038/nmeth0411-304 21451519

[32] Starr R, Stahlheber S, Small A (2012) Fast maximum likelihood algorithm for localization of fluorescent molecules. Opt Lett 37(3): 413–415. 22297370 10.1364/OL.37.000413

[33] Zhu L, Zhang W, Elnatan D, Huang B (2012) Faster STORM using compressed sensing. Nat Methods 9(7): 721–723. 22522657 10.1038/nmeth.1978 PMC3477591

[34] Mukamel EA, Babcock H (2012) Zhuang× (2012) Statistical deconvolution for superresolution fluorescence microscopy. Biophys J 102(10): 2391–2400. 22677393 10.1016/j.bpj.2012.03.070 PMC3353000

[35] Smith CS, Joseph N, Rieger B, Lidke KA (2010) Fast, single-molecule localization that achieves theoretically minimum uncertainty. Nat Methods 7(5): 373–375. 20364146 10.1038/nmeth.1449 PMC2862147

[36] Diaspro A, Chirico G, Collini M (2006) Two-photon fluorescence exitation and related thechniques in biological microscopy. Q Rev Biophys 15: 1–70. 10.1017/S0033583505004129 16478566

[37] Post JN, Lidke KA, Rieger B, Arndt-Jovin DJ (2005) One- and two-photon photoactivation of a paGFP-fusion protein in live Drosophila embryos. FEBS Letters 579: 325–330. 15642339 10.1016/j.febslet.2004.11.092

[38] Schneider M, Barozzi S, Testa I, Faretta M, Diaspro A (2005) Two-photon activation and excitation properties of PA-GFP in the 720–920-nm region. Biophys J 89(2): 1346–1352. 15908572 10.1529/biophysj.104.054502 PMC1366619

[39] Vaziri A, Tang J, Shroff H, Shank CV (2008) Multilayer three-dimensional super resolution imaging of thick biological samples. Proc Natl Acad Sci 105(51): 20221–20226. 19088193 10.1073/pnas.0810636105 PMC2603430

[40] Fölling J, Belov V, Riedel D, Schönle A, Egner A, et al (2008) Fluorescence nanoscopy with optical sectioning by two-photon induced molecular switching using continuous-wave lasers Chemphyschem. 9(2): 321–326. 10.1002/cphc.200700655 18200483

[41] Mazza D, Cella F, Vicidomini G, Krol S, Diaspro A (2007) Role of three-dimensional bleach distribution in confocal and two-photon fluorescence recovery after photobleaching experiments Appl Opt. 46(30): 7401–7411. 10.1364/ao.46.007401 17952174

[42] Mourant JR, Freyer JP, Hielscher AH, Eick AA, Shen D, et al (1998) Mechanisms of light scattering from biological cells relevant to noninvasive optical-tissue diagnostics. Appl Opt 37(16): 3586–3593. 18273328 10.1364/ao.37.003586

[43] Fölling J, Belov V, Kunetsky R, Medda R, Schönle A, et al (2007) Photochromic rhodamines provide nanoscopy with optical sectioning. Angew Chem Int Ed Engl 46(33): 6266–6270. 17640007 10.1002/anie.200702167

[44] Mortensen KI, Churchman LS, Spudich JA, Flyvbjerg H (2010) Optimized localization analysis for single-molecule tracking and super-resolution microscopy. Nat Methods 7(5): 377–381. 20364147 10.1038/nmeth.1447 PMC3127582

[45] Lanotte M, Martin-Thouvenin V, Najman S, Balerini P, Valensi F, et al (1991) NB4, a maturation inducible cell line with t(15;17) marker isolated from a human acute promyelocytic leukemia (M3) Blood. 77(5): 1080–1086. 1995093

