# Correction: Light-Sheet Confined Super-Resolution Using Two-Photon Photoactivation

**DOI:** 10.1371/annotation/87ab5def-8daf-4c15-ba52-8fc1d5b8a333

**Published:** 2013-10-24

**Authors:** Francesca Cella Zanacchi, Zeno Lavagnino, Mario Faretta, Laura Furia, Alberto Diaspro

There were multiple omissions of multiple authors' additional contributions to the article in the Author Contributions Statement. The following should be included in the Author Contributions Statement: "Conceived research: A.D., F.C.Z.

Discussed the conceptual and practical implications of the method at all stages: F.C.Z., A.D., Z.L., M.F., L.F." 

